# Kinetics and Isotherm Study of Ceftriaxone Removal Using Functionalized Biochar Combined with Photocatalysis

**DOI:** 10.3390/molecules30214291

**Published:** 2025-11-05

**Authors:** Luísa Cruz-Lopes, Rodrigo Araújo, Ana Rita Lopes, Samuel Moles, Francisca Romero-Sarria, Bruno Esteves

**Affiliations:** 1 CERNAS (Centre for Natural Resources, Environment and Society)-IPV Research Centre, Polytechnic University of Viseu, Av. Cor. José Maria Vale de Andrade, 3504-510 Viseu, Portugal; lvalente@estgv.ipv.pt; 2 Department of Environmental Engineering, Polytechnic University of Viseu, Av. Cor. José Maria Vale de Andrade, 3504-510 Viseu, Portugal; rodrigokenzo2202@gmail.com; 3 Faculty of Dental Medicine, Universidade Católica Portuguesa, 3504-505 Viseu, Portugal; s-arvlopes@ucp.pt; 4 Instituto de Investigación en Ciencias Ambientales de Aragón (IUCA), Universidad de Zaragoza, c. de Pedro Cerbuna, 12, 50009 Zaragoza, Spain; sma@unizar.es; 5 Department of General Chemistry, Escuela Universitaria Politécnica La Almunia Universidad de Zaragoza, C. Mayor, 5, La Almunia de Doña Godina, 50100 Zaragoza, Spain; 6 Department of Inorganic Chemistry and Institute of Materials Science, Joint Center University of Seville-CSIC, Av. Américo Vespucio, 41092 Seville, Spain; francisca@us.es

**Keywords:** antibiotic, ceftriaxone sodium, cephalosporins, biochar, photocatalysis, wastewater treatment

## Abstract

The increasing presence of antibiotics such as cephalosporins in wastewater represents a significant environmental risk. These compounds are excreted in large quantities, and conventional wastewater treatment plants are often ineffective at their removal. Consequently, the development of more sustainable and efficient treatment technologies is essential. In this study, the removal of cephalosporins from aqueous solutions was evaluated through adsorption using pine bark biochar, photocatalysis with TiO_2_, and a combination of both processes. Kinetic experiments were conducted with cephalosporin solutions (15 mg/L), employing 150 mg/L of biochar, 100 mg/L TiO_2_, or their combination, under continuous stirring and/or UV-vis irradiation. Samples were collected at 0 and 120 min and analyzed via UV-vis spectrophotometry. Adsorption isotherms were established for initial cephalosporin concentrations ranging from 5 to 50 mg/L. The biochar alone achieved a removal efficiency of 94.2% after 120 min. Photocatalysis with TiO_2_ alone resulted in 75% removal, while the combined approach reached 95.9%, indicating a synergistic effect between adsorption and photodegradation mechanisms. Kinetic data fitted the pseudo-second-order model, and the Langmuir isotherm provided the best correlation, suggesting monolayer adsorption. These findings demonstrate that pine bark biochar, whether used independently or in combination with TiO_2_, constitutes an eco-friendly, effective, and low-cost alternative for the removal of antibiotics from wastewater, while simultaneously contributing to the valorization of forestry residues.

## 1. Introduction

In recent years, the growing expansion of the pharmaceutical sector has led to a marked increase in the consumption of antibiotics for the treatment of various diseases, resulting in their frequent detection in aquatic environments [1,2,3].The continuous release of these compounds, particularly into drinking water sources, poses a significant risk to water quality, aquatic ecosystems, and public health [4]. Antibiotics are often only partially metabolized by humans and animals, with estimates indicating that between 30% and 90% of the administered doses are excreted unchanged via urine and feces [5]. Additional sources of environmental contamination include emissions from pharmaceutical manufacturing processes, inadequate disposal of unused medications, and the release of effluents into sewage systems [6,7]. Furthermore, studies have consistently demonstrated that conventional municipal wastewater treatment plants (WWTPs) are not fully effective in eliminating pharmaceutical residues, allowing these micropollutants present at very low concentrations to persist in treated effluents. For instance erythromycin degradation products, along with roxithromycin and sulfamethoxazole, were frequently detected in the examined sewage treatment plants effluents and surface water samples, reaching concentrations of up to 6 μg/L. [8]. According to Moles et al. [9], antibiotics are frequently detected in water bodies at concentrations spanning from ng/L to μg/L, with sulfonamides, trimethoprim, β-lactams, and fluoroquinolones being among the most prevalent classes reported. Given that conventional WWTPs are not specifically designed to remove antibiotic microcontaminants, it has become crucial to investigate advanced treatment strategies such as ozonation, ultraviolet irradiation, chlorination, nanofiltration, and reverse osmosis [10]. Despite their effectiveness, these techniques are often associated with high operational and maintenance costs, especially when applied to large volumes of wastewater. In this context, photocatalysis and adsorption have emerged as promising, cost-effective, and environmentally friendly alternatives for the removal of pharmaceutical compounds [9]. In particular, the use of natural materials such as biochar, produced from lignocellulosic biomass, has gained increasing attention [2,11].

Biochar is a carbonaceous material obtained by pyrolysis of organic biomass—including agricultural residues, forestry by-products, and livestock waste—under oxygen-limited conditions at temperatures typically ranging from 350 °C to 700 °C [12,13]. Production conditions and post-treatments are selected to tailor surface area, pore structure and surface chemistry for antibiotic adsorption purposes. Reviews and recent syntheses emphasize conventional pyrolysis (slow/fast), hydrothermal carbonization, microwave treatment, gasification, and torrefaction and a wide range of physical/chemical activation or metal/magnetic/biopolymer modifications to create “designer” biochars with enhanced adsorption or catalytic functions [14]. Performance is compound- and material-dependent. The choice of biomass feedstock and the conditions applied during pyrolysis strongly influence the physicochemical properties of biochar, including its surface area and pore architecture (micropores < 2 nm, mesopores 2–50 nm, macropores > 50 nm), which in turn determine its functional applications. Macropores primarily facilitate substance diffusion, mesopores serve as channels for mass transfer, and micropores provide sites for adsorption and entrapment [15]

Production and modification of biochar involve several key factors with practical implications. Pyrolysis variables such as temperature and heating rate play a central role in determining aromaticity, the presence of oxygen-containing functional groups, and porosity. In general, biochar produced under higher pyrolysis temperatures exhibits improved performance in removing organic contaminants due to increased surface area, enhanced hydrophobicity, and greater microporosity [16]. On the other hand lower temperatures can help preserve polar functional groups [17]. Activation routes, including physical methods (steam, CO_2_), chemical treatments (KOH, H_3_PO_4_, acids/bases), and ball milling, are commonly used to increase accessible surface area and generate micro- and mesopores [18,19].

In addition, composite and targeted designs have been developed; for example, magnetic materials (Fe/Zn, Fe_3_O_4_), metal-impregnated forms, chitosan/biochar composites, and MOF (Metal–Organic Framework)-derived biochars produce materials that are easier to separate or combine adsorption with catalytic degradation [20,21,22]. The choice of feedstock, whether agricultural residues, sewage sludge, spent coffee grounds, or forestry residues, directly affects ash content and surface chemistry, thereby influencing the affinity of biochar for specific antibiotics [18,20,23]

Antibiotic uptake onto biochar is governed by multiple simultaneous physicochemical interactions whose relative importance depends on the biochar surface and the antibiotic’s chemistry. Several studies identify electrostatic attraction/repulsion, hydrophobic partitioning, π–π electron donor–acceptor interactions, hydrogen bonding, surface complexation/coordination and pore filling as primary mechanisms [24]. Electrostatic interactions are particularly important, as they depend on solution pH and the relationship between the biochar point of zero charge and antibiotic ionization constants (pKa), often controlling the sorption of ionizable antibiotics [13]. Hydrophobic partitioning and π–π interactions dominate in the case of aromatic or planar antibiotics such as fluoroquinolones and macrolides, which interact strongly with graphitic domains on biochar, especially those produced at high temperatures with high aromaticity [17,24].

Hydrogen bonding and surface complexation also play a role, involving interactions between polar functional groups on biochar (–OH, –COOH, heteroatoms) and polar moieties of antibiotics such as tetracyclines and sulfonamides [13,19]. In cephalosporins hydrogen bonding and π–π interactions enhance adsorption, by promoting binding to functional groups and aromatic domains on the biochar surface [25]. Pore filling and size exclusion are key factors, as the micro- and mesopore volume and pore size distribution influence both adsorption capacity and kinetics. The porous structure of biochar facilitates pore diffusion, enabling antibiotic molecules to penetrate its internal network and increasing overall adsorption rates [26].

Different antibiotics show varying adsorption and removal efficiencies when treated with biochars and their engineered composites. Ciprofloxacin (CIP) demonstrates very high adsorption capacities in engineered biochars, with reports of values around 550 mg·g^−1^ for CAB (alkali activated pyrolysis), and has shown effective removal across multiple studies [20,27]. Sulfamethoxazole (SMX) generally exhibits moderate to high removal, with reported efficiencies ranging from over 30% to 80% depending on the specific biochar type and environmental matrix [17,19]. Tetracycline (TET) is strongly adsorbed by magnetic and metal-doped biochars, and high adsorption capacities are frequently reported when using engineered composites [20,22]. Macrolides such as clarithromycin and erythromycin have been shown to undergo high removal in hospital effluent experiments and sorbent blends, with clarithromycin reduced to low nanogram-per-liter concentrations [17,23]. Trimethoprim removal has also been highly effective in hospital wastewater experiments, with reported efficiencies of approximately 91% [17]. By contrast, certain biochars have been less effective in removing ofloxacin or have shown poor SMX removal in feedstock-specific studies [13].

Reported adsorption capacities and percentage removals vary considerably depending on factors such as the initial antibiotic concentration, contact time, adsorbent dose, and the presence of competing constituents in real wastewater, as a result, direct comparisons across studies are only meaningful when experimental conditions are closely matched [17,23]. Modifications including magnetic functionalization, metal doping, chitosan composites, ball milling, and chemical activation have frequently been shown to enhance the removal of target antibiotics compared to pristine biochar [19,20,21].

Among its main advantages, biochar provides cost-effectiveness and sustainability, as it can be produced from wastes and residues, aligning with circular economy principles [23]. Its tunability is another benefit, since production and modification strategies allow tailoring to specific antibiotics through approaches such as magnetic separation, introduction of metal sites, or incorporation of polymer composites [14,21]. Biochar also offers multifunctionality, combining adsorption with catalytic oxidation or enabling magnetic separation when doped with metals [20,22]. In addition, regeneration potential is increasingly recognized, with electrothermal and carbothermal shock approaches showing promise for rapid, energy-efficient regeneration and reuse [27].

The use of biochar for the removal of cephalosporins, such as cefixime and cephalexin, has shown promising results in various studies [25,28]. Biochar, derived from organic materials, exhibits high adsorption capacities due to its porous structure and surface functional groups, making it an effective adsorbent for antibiotics in aqueous solutions. The effectiveness of biochar for cephalosporin removal is evident from reported adsorption capacities, which reach up to 21.51 mg/g for cefixime with biochar prepared from pine leaves (*Pinus kesiya*) and 57.47 mg/g for cephalexin with biochar obtained from palm oil fiber, demonstrating substantial potential for efficient antibiotic removal. Adsorption kinetics generally follow a pseudo-second-order model, indicating that the rate of adsorption is governed by the availability of active binding sites on the biochar surface [29]. In practical applications, biochar has been successfully tested in synthetic wastewater matrices, showing its capacity to remove trace antibiotics and providing a sustainable approach for environmental remediation [25]. Its production from agricultural waste further enhances cost-effectiveness, offering a low-cost alternative for treating antibiotic-contaminated water [28,29].

Pine bark biochar has been used for the removal of several antibiotics. For example, iron-coated pine bark biochar (Fe-PBB) was used to remove tetracycline from waste waters and a 95% efficiency was obtained for the concentration of 1 mg/L and a Fe-PBB dose of 2 g/L at pH 7 and 50 °C [30]. Biochar derived from Pinus kesiya bark through microwave-assisted pyrolysis demonstrated a maximum ciprofloxacin removal efficiency of 90.97% under optimal conditions of pH 6, an initial concentration of 20 mg/L, and an equilibrium time of 180 min [31]. In the study by Naghipour et al. [32] where pine bark biochar was used, the maximum tetracycline removal efficiency of 89.5% was achieved within 15 min at pH 5.1 and an adsorbent dose of 1 g. The researchers found that the removal efficiency increased with higher temperatures and greater adsorbent doses, while it declined with increasing contaminant concentrations.

Titanium dioxide (TiO_2_) has been studied before for the removal of antibiotics from aqueous systems due to its dual role as an adsorbent and photocatalyst. The adsorption step is crucial, as it concentrates antibiotic molecules near the TiO_2_ surface, where surface hydroxyl groups, electrostatic interactions, and ligand exchange with carboxylate moieties enhance binding. This pre-adsorption facilitates subsequent photocatalytic degradation. Upon irradiation with light of energy equal to or greater than its band gap, TiO_2_ generates electron–hole pairs that lead to the formation of reactive oxygen species, primarily hydroxyl radicals (OH) and superoxide radicals (O_2_^−^). These oxygen species attack antibiotic functional groups such as β-lactam rings, existing in cephalosporins, amino substituents, and aromatic moieties, resulting in progressive degradation and eventual mineralization [33]. However, efficiency depends on several factors, including pH, pollutant concentration, catalyst dosage, and the presence of inorganic ions or natural organic matter. Modifications to TiO_2_, such as doping or coupling with other semiconductors, are explored to extend photoactivation into the visible spectrum and reduce charge recombination, thereby enhancing photocatalytic efficiency. The photocatalytic pathways involve hydroxylation, demethylation, defluorination, and ring cleavage, producing intermediate compounds whose ecotoxicity must be carefully assessed, since some byproducts can be more toxic than the parent molecules [34].

The synergy between adsorption and photocatalysis makes TiO_2_ an efficient material for antibiotic removal, though limitations such as UV dependence and electron–hole recombination have prompted efforts to improve its performance through doping, sensitization, or coupling with carbon-based supports [33]. The study by Nezhal et al. [35] used MgO nanoparticles to enhance the photocatalytic performance of TiO_2_ and demonstrated that TiO_2_/MgO nanoparticles achieved a ceftriaxone sodium removal efficiency of 93.7% from synthetic wastewater at a concentration of 400 mg/L under ultraviolet radiation, confirming the effectiveness of this photocatalyst in removing the antibiotic. Similarly, another study used a 500 mg/L solution, even higher but achieved a degradation degree of 96.52% under optimal conditions: 100 mg TiO_2_ nanoparticles, pH 8, and 9 h of radiation with a white mercury UV lamp, resulting in non-toxic degradation products [36].

This study aims to develop and evaluate a sustainable and efficient treatment method for removing cephalosporin (ceftriaxone sodium) antibiotics from wastewater. It focuses on using pine bark biochar, TiO_2_ photocatalysis, and their combination to enhance removal efficiency through adsorption and photodegradation mechanisms. It is expected that biochar can improve the adsorption of ceftriaxone sodium that can be latter degraded by photocatalyzed TiO_2_. By exploring kinetic and isotherm models, the research seeks to understand the interaction between cephalosporins and the treatment materials, highlighting pine bark biochar as a low-cost, eco-friendly solution that also promotes the valorization of forestry residues.

## 2. Results and Discussion

### 2.1. Biochar Production and Characterization

The choice of using lignocellulosic materials such as pine bark is based on their environmentally friendly nature, as they do not present harm to the environment. Additionally, they represent a sustainable and cost-effective option, given their low cost and widespread availability [2]. The material in question was converted into its biochar form through a process known as pyrolysis, which involves the thermal decomposition of biomass in the absence (or under limited supply) of oxygen. Biochar exhibits multilevel structural complexity that enables the occurrence of multiple sorption mechanisms. Studies have also demonstrated that modifications to biochar, such as coating with humic acid, can enhance these interactions, particularly in the adsorption of antibiotics [37].

The characterization of the biochar is presented in Table 1.

The results clearly demonstrate that the CO_2_-activated biochar exhibits good physicochemical characteristics specifically its high specific surface area, and a developed microporous and mesoporous structure. These properties significantly enhance the material’s potential for applications in pollutant adsorption from aqueous media, making the CO_2_-activated biochar a suitable material for environmental remediation purposes.

FTIR characterization (Figure S1) reveals significative bands at 1700, 1600, 1450, 900–700 cm^−1^. The FTIR spectrum of the CO_2_-activated biochar displays the expected fingerprint of a predominantly aromatic carbon matrix with minor oxygenated functionalities. This aligns with other studies concerning biochar activation [38]. A weak feature near ~1700–1680 cm^−1^ is consistent with residual C=O stretching from carbonyl/ketone groups, while a clear band at ~1600 cm^−1^ corresponds to aromatic C=C stretching, evidencing condensed/graphitic domains. An additional band around ~1450 cm^−1^ indicates the presence of carbonate species (ν_3_, CO_3_^2−^) associated with the mineral fraction after CO_2_ activation and partially overlapping with nearby C–H deformation modes. The broad region ~1230–1050 cm^−1^ arises from C–O and C–O–C vibrations of phenolic/ether/alcohol groups that remain on the surface. Finally, the strong multiplet between ~900 and 700 cm^−1^, assigned to out-of-plane =C–H bending of aromatic rings [39], supports a high degree of polyaromatic condensation. According to the literature, this activation procedure can leave condensed aromatics while promoting carbonate signals from ash/mineral fractions [40]

An adsorbent with a specific surface area of 583 m^2^/g and a pore structure dominated by micropores (less than 2 nm) and mesopores (2–50 nm) has several notable characteristics [41]. SEM images of the biochar (Figure S2) display a highly porous texture characterized by abundant meso- and micropores, consistent with the development of fine porosity during thermal treatment. The high surface area is typical of materials such as activated carbons, zeolites, or certain metal–organic frameworks and generally correlates with a high adsorption capacity, particularly for gases and small molecules. Even though the surface area is in the low end compared to activated carbons (500–3000 m^2^/g) it shows a specific surface area higher than most biochar [42]. Compared to previously reported pine bark-based biochar [32] (specific surface area 683 m^2^/g, pHpzc 7.5), the prepared biochar exhibited a lower surface area (583 m^2^/g) and slightly more acidic surface properties (pHpzc 6.7), likely reflecting differences in preparation conditions.

Each pore type contributes differently to adsorption. Macrospores primarily support substance diffusion, while mesopores function as channels for mass transfer, and micropores serve as sites for molecular entrapment. Micropores account for most of the surface area, making them especially effective for adsorbing small molecules such as CO_2_, CH_4_, H_2_, and volatile organic compounds, and they dominate adsorption at low relative pressures. In contrast, mesopores enhance diffusion and accessibility by acting as transport pathways that link micropores and improve adsorption kinetics [41,42,43].

The micropores provide most of the surface area and are ideal for adsorbing small molecules such as CO_2_, CH_4_, H_2_, or volatile organic compounds. They dominate adsorption at low relative pressures. Mesopores, on the other hand, improve diffusion and accessibility, serving as transport channels that connect micropores and enhance adsorption kinetics.

Overall, a 583 m^2^/g adsorbent with predominantly micropores and mesopores is highly promising for gas adsorption, separation, and catalytic applications, effectively balancing storage capacity with transport efficiency.

XRD patterns of biochar are presented in Figure S4. The thermal decomposition of calcium carbonate typically occurs around 825 °C; however, this process is inhibited in a CO_2_-rich atmosphere as can be seen in Figure S4. In addition, two broad diffraction bands observed at approximately 18–28° and 42–47° (2θ) are indicative of carbonaceous structures (Figure S4). The first reflection corresponds to the (002) crystallographic plane and provides information about the alignment of aromatic layers generated during pyrolysis—the sharper this signal, the greater the structural ordering. The second reflection, associated with the (101) plane, reflects the lateral size of the aromatic domains; a more intense and defined peak denotes a higher degree of aromatic condensation.

### 2.2. pH Optimization

**Figure 1:**
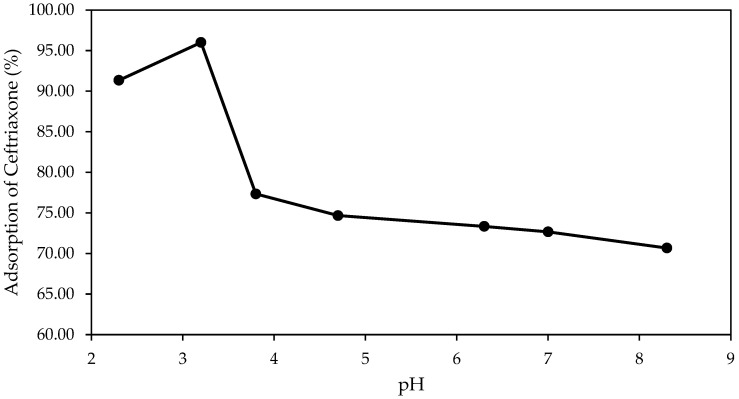
Adsorption of CEF onto pine bark biochar at different initial pH values after 60 min of contact (initial concentration: 15 mg/L; biochar dosage: 150 mg/L; pH range: 2–9).

Figure 1 shows the effect of the initial pH on the adsorption efficiency of CEF after 60 min of contact time (initial concentration: 15 mg/L; pine bark biochar dosage: 150 mg/L; pH range: 2–9).

The adsorption of CEF onto the biochar was found to be highly dependent on the solution pH, with maximum adsorption occurring at around pH 3, where removal exceeded 95%.

The CEF molecule possesses multiple ionizable groups, with reported pKa values of approximately 2.4 (carboxyl), 3.0 (aminothiazole), 4.2–5.6 (hydroxytriazinone), and 10.7 (amide) [44]. Other authors refer different values but more or less in the same range, like (pKa_1_ = 3.0, pKa_2_ = 3.2 and pKa_3_ = 4.1) [45]. According to Zheng et al. [46] CEF has six acid dissociation constants (pKa values) at 2.6, 3.0, 3.2, 3.9, 4.6, and 10.7.

At highly acidic pH, the CEF molecule exists in a cationic state with a net charge of +1. This is because the aminothiazole group (pKa 3.0) is protonated, while the other groups are in their neutral, protonated forms. As the pH rises above 2.4, the carboxyl group deprotonates, creating a negative charge. In the narrow range between pH 2.4 and 3.0, the molecule is zwitterionic, carrying both a positive charge from the protonated aminothiazole and a negative charge from the deprotonated carboxyl group, resulting in a net neutral charge. Above pH 3.0, the aminothiazole group deprotonates to a neutral state. With the carboxyl group already deprotonated, the molecule now has a net negative charge, making it anionic. This anionic character becomes more pronounced as the pH increases further, causing the hydroxytriazinone (pKa 4.2–5.6) and finally the amide (pKa 10.7) groups to deprotonate, leading to increasingly negative charges. Since the biochar has a point of zero charge (PZC) of 6.7 this means it will have a net positive surface charge at a pH below 6.7 and a net negative surface charge at a pH above 6.7. CEF, as described, is anionic at a pH above 3.0. Therefore, the biochar is expected to adsorb CEF most effectively above pH 3 and below its PZC of 6.7, and specifically within a pH range where the CEF molecule has a negative charge and biochar positive. It is more likely that the final pH, after adding the biochar, will be higher than the initial measured pH, possibly increasing to a range of 4–5. This pH is ideal because it is where CEF exists as an anionic molecule, while the biochar remains cationic, promoting optimal adsorption due to electrostatic attraction. If we consider the 7 pKa values reported before the discussion still applies. Below pH 3.0, CEF exists predominantly in its positively charged form (CEF^+^). In the pH range of 3.0–3.2, it mainly occurs as a mixture of the zwitterionic species and the neutral form and at pH values above 4.6, CEF is largely present as negatively charged species, including CEF^−^, CEF^2−^, and CEF^3−^ [46].

These findings confirm that pH significantly influences the adsorption process by affecting both the surface charge properties of the biochar and the ionization state of the antibiotic molecules [47].

**Figure 2:**
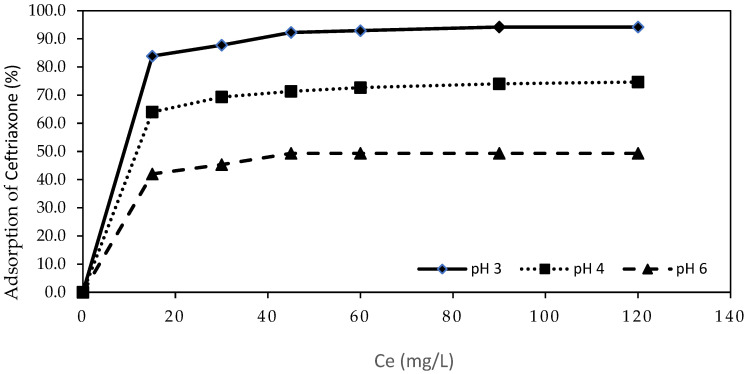
CEF adsorption efficiency at initial pH 3, 4, and 6 after 120 min (15 mg/L CEF; 150 mg/L Pine bark biochar).

Figure 2 presents the adsorption kinetics of CEF at different pH values (pH 3, 4, and 6) over a 120 min period.

Adsorption was found to be more effective under acidic conditions, with the highest adsorption rate observed at initial pH 3, resulting in a removal efficiency of 94.2% after 120 min (Figure 2). The kinetic profiles reveal that the highest retention of CEF is achieved within the first 15 min. At pH 4 and 6, the adsorption process is comparatively slower, and the equilibrium adsorption capacity is lower. This can be explained by the decrease in electrostatic attraction at higher pH values, as the surface of the biochar becomes less positively charged and the CEF species exist in more neutral or zwitterionic forms, reducing interaction efficiency.

Moreover, the initial rapid adsorption phase followed by a slower approach to equilibrium, observed at all pH values, suggests that the process is initially controlled by external surface adsorption, while at later stages intraparticle diffusion becomes more significant.

Thus, both Figure 1 and Figure 2 collectively highlight the crucial role of pH in determining not only the final adsorption efficiency but also the kinetics of CEF removal using pine bark-derived biochar.

Pine bark biochar has been used for the removal of different antibiotics such as, for example, tetracycline with the highest adsorption efficiency (90%) obtained for pH 5.1 [32].

The study by Berges et al. [1] that studied antibiotics removal from aquatic environments on vegetal powdered activated carbon, presented similar results, showing that sorption was more efficient at lower pH values for both sulfadiazine and amoxicillin, which are β-lactam antibiotics, similar to CEF. This enhanced sorption occurs due to the establishment of hydrogen bonds and the presence of a charged carboxyl group in these compounds, respectively. On the other end CEF biosorption by *Pseudomonas putida* was found to be maximum at pH 7 [48] similar to the adsorption using mesoporous copper oxide nanospheres [49].

### 2.3. Adsorption Isotherm

The adsorption isotherms were established by evaluating the adsorption of CEF at increasing initial concentrations, maintaining a constant amount of adsorbent and a fixed temperature. The experimental data were analyzed using both the Langmuir and Freundlich models, which are widely employed to describe adsorption phenomena on homogeneous and heterogeneous surfaces, respectively.

**Figure 3:**
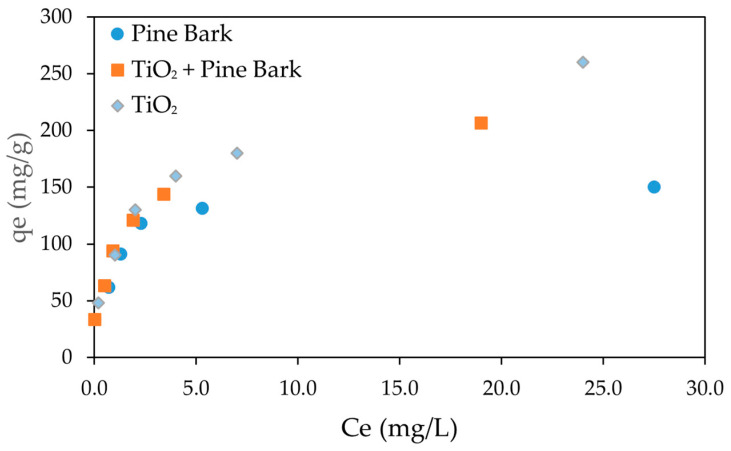
Adsorption isotherm of CEF on pine bark biochar and TiO_2_. Ce represents the equilibrium concentration (initial CEF concentrations ranging from 5 to 50 mg/L; biochar dosage: 150 mg/L; TiO_2_ dosage = 100 mg/L); adsorption time: 120 min.

Figure 3 presents the adsorption isotherms of CEF onto pine bark biochar, TiO_2_, and the combination of pine bark biochar with TiO_2_, using initial CEF concentrations ranging from 5 to 50 mg/L.

The adsorption isotherms exhibited a typical Type I behavior for all adsorbents, characterized by a rapid initial increase in adsorption capacity (*q_e_*) at low equilibrium concentrations (*C_e_*), followed by a gradual plateau as *C_e_* increased. This behavior suggests that adsorption initially occurs rapidly due to the abundance of available active sites, progressively slowing as the surface becomes saturated, consistent with monolayer adsorption. This Type I isotherm is expected according to IUPAC classification if micropores strongly dominate, showing sharp uptake at low relative pressures and a plateau at higher pressures. If mesoporosity is significant, a Type IV isotherm may appear, with a hysteresis loop due to capillary condensation in mesopores.

Among the tested systems, TiO_2_ showed the highest adsorption capacity across the concentration range, while the combination of pine bark biochar with TiO_2_ also exhibited high performance, though slightly lower, indicating a synergistic effect between adsorption and photocatalytic processes. Pine bark biochar alone demonstrated a good adsorption capacity, although lower than the other two systems. The results indicate that adsorption equilibrium was achieved at an initial concentration of approximately 50 mg/L which corresponded to an equilibrium concentration of about 20 mg/L for TiO_2_ + pine bark biochar, 25 mg/L for TiO_2_ and 27 mg/L for pine bark biochar. in all experimental conditions. These findings highlight the relevance of adsorbent properties in determining adsorption performance and support the modeling of the process using the Langmuir and Freundlich isotherms.

**Figure 4:**
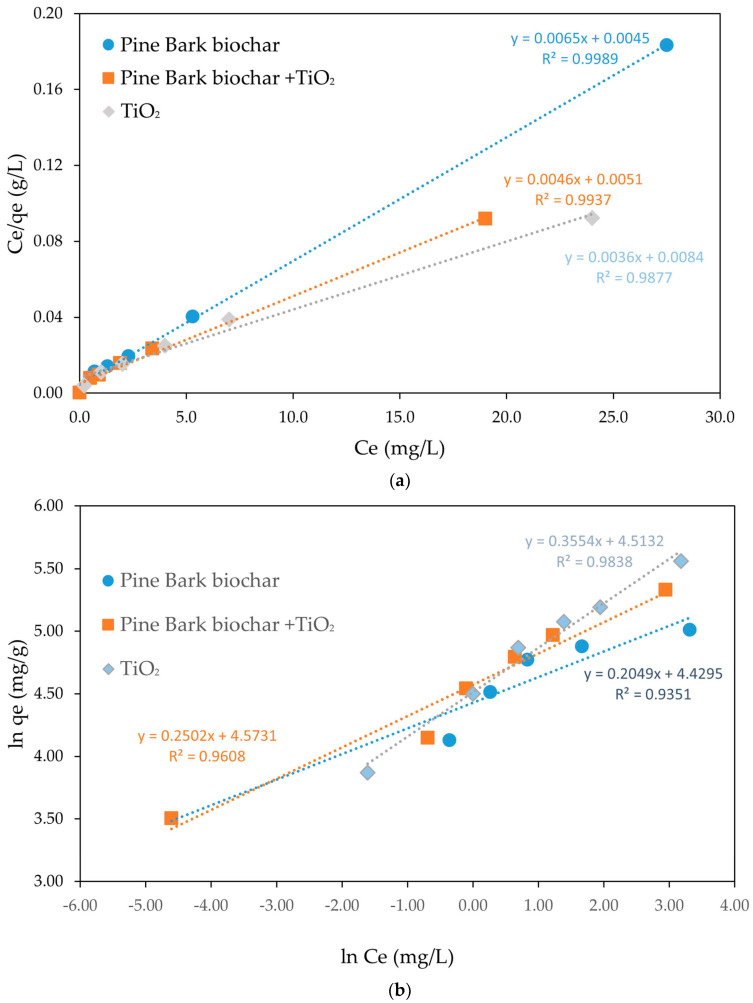
Linearized isotherm models for CEF adsorption onto pine bark biochar, TiO_2_, and pine bark biochar + TiO_2_ where Ce represents the equilibrium concentration (initial CEF concentrations: 5–50 mg/L; adsorbent dosage: 150 mg/L): (**a**) Langmuir; (**b**) Freundlich.

The results derived from the linearized representations of the Langmuir and Freundlich equilibrium isotherms are shown in Figure 4a,b and summarized in Table 2. Figure 4 shows the linearized Langmuir adsorption isotherms for CEF adsorption onto pine bark biochar, TiO_2_, and the combination of pine bark biochar with TiO_2_. A strong linear relationship between *C_e_/q_e_* and *C_e_* was observed for all adsorbents, confirming that the adsorption process follows Langmuir model assumptions, characterized by monolayer adsorption onto homogeneous surfaces without interaction between adsorbed molecules. Among the materials, pine bark biochar (R^2^ = 0.999) exhibited the highest linearity, indicating the most homogeneous surface and strongest conformity to the Langmuir model, followed by the combination of pine bark biochar with TiO_2_ (R^2^ = 0.994) and TiO_2_ alone (R^2^ = 0.988). The steep slopes of the plots suggest a high affinity between CEF molecules and the adsorbent surfaces, particularly in the combined system. Overall, the results confirm that the adsorption of CEF onto the tested materials occurs predominantly through monolayer formation, with the combined biochar and TiO_2_ system exhibiting enhanced adsorption properties. The Langmuir model assumes the formation of a monolayer and a uniform distribution of adsorption sites across the surface of the adsorbent [1]. The model is particularly useful when there is a strong and specific interaction between the adsorbent surface and the adsorbate, leading to the formation of a single adsorbed layer, with no occurrence of multilayer adsorption [50]. It additionally suggests that there are no interactions between adsorbed molecules, and that adsorption occurs under isothermal conditions on a homogeneous surface.

Figure 4b shows the linearized Freundlich adsorption isotherms for CEF adsorption onto pine bark biochar, TiO_2_, and the combination of pine bark biochar with TiO_2_. The plots display a reasonable but less pronounced linear relationship between ln(*q_e_*) and ln(*C_e_*), indicating that adsorption occurs on heterogeneous surfaces with multilayer formation. Among the materials, TiO_2_ showed the best linear fit (R^2^ = 0.984), followed by the combination of pine bark biochar and TiO_2_ (R^2^ = 0.961), and pine bark biochar alone (R^2^ = 0.935), suggesting greater surface heterogeneity in the biochar samples.

Overall, the results suggest that, although surface heterogeneity might be present, the adsorption of CEF is mainly governed by monolayer mechanisms, as better described by the Langmuir model. Similar results were presented before for several different adsorbents such as using mesoporous copper oxide nanosphere where Langmuir model had a R^2^ = 0.999 much higher than the Freundlich model (R^2^ = 0.774) [49], using a combined electrocoagulation and absorption with chitosan [51] or tea waste activated carbon [52]. Most of the studies on the adsorption of antibiotics onto pristine biochar are described by the Langmuir isotherm, suggesting monolayer adsorption on uniform surfaces [42]. This happens not only for CEF but also for other groups of antibiotics. For instance, the adsorption of tetracycline antibiotics onto wheat-stalk biochar fitted well with the Langmuir model [53], and similar trends were reported for sulfonamide antibiotics, including sulfadiazine (SDZ) and sulfamethoxazole (SMX) [18].

The Freundlich model under isothermal conditions, assumes that adsorption occurs on a heterogeneous surface with a non-uniform distribution of adsorption heat and active sites possessing varying energy levels [2]. However, this model is typically applicable over a limited concentration range. When fitted to experimental data at high and intermediate concentrations, it often fails to accurately represent adsorption behavior at low concentrations [50].

Table 2 summarizes the parameters obtained from the linearized Langmuir and Freundlich isotherm models for CEF adsorption onto pine bark biochar, TiO_2_, and the combined system. The adsorption of CEF onto pine bark biochar, TiO_2_, and their combination follows predominantly a Langmuir-type behavior, as stated before.

TiO_2_ showed the highest adsorption capacity of 280 mg/g and Pine bark biochar alone the lowest (154 mg/g). Nevertheless, all the values are higher than the adsorption capacity obtained by using mesoporous copper oxide nanosphere (127 mg/g) [49] or combined electrocoagulation and absorption with chitosan (111.1 mg/g) [51] for CEF adsorption. These values could not reach however the capacity of 787.5 mg/g reported by using tea waste activated carbon [52].

The R_L_ parameter, derived from the Langmuir isotherm, is an important indicator of adsorption favorability. For the adsorption of CEF onto pine bark biochar, the calculated R_L_ value was 0.01 L/mg. Since R_L_ values between 0 and 1 indicate favorable adsorption, and values closer to 0 suggest a highly favorable process, this result demonstrates that pine bark biochar possesses a strong affinity for CEF molecules. The very low R_L_ value highlights the efficiency of the biochar surface in retaining CEF, suggesting its potential as an effective adsorbent for removing pharmaceutical contaminants from aqueous solutions. The TiO_2_ R_L_ values suggest that adsorption by this material is also favorable but less than Pine bark Biochar and that, as expected, the mixture of both adsorbents has an intermeddiate value. The same can be seen by the 1/n parameter from Freudlich isotherm since it provides insight into the adsorption intensity and surface heterogeneity of an adsorbent. For the adsorption of CEF onto pine bark biochar, the 1/n value was found to be 0.20 which also indicates favorable adsorption, reflecting stronger adsorption affinity promoting strong binding interactions, supporting the potential of pine bark biochar as an efficient material for removing pharmaceutical contaminants from water. Similarly to R_L_ for TiO_2_ the value is higher and for the mixture is between the two.

### 2.4. Kinetics Studies

**Figure 5:**
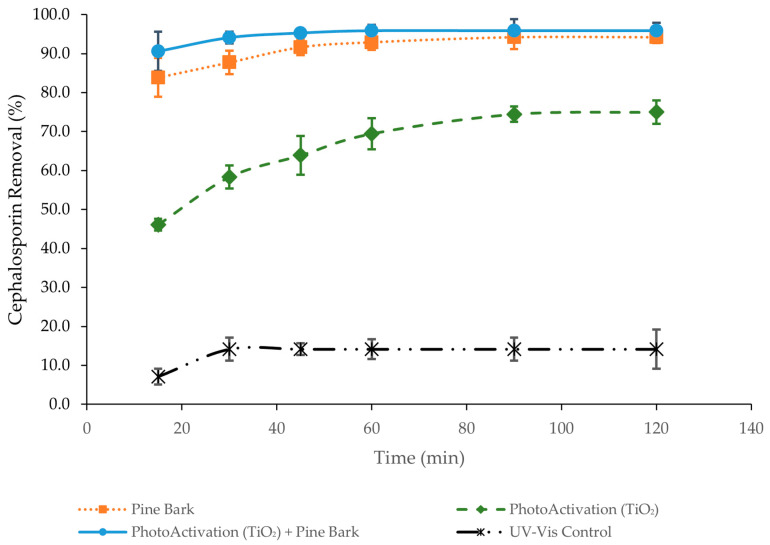
Adsorption efficiency of CEF versus time (15 to 120 min) using pine bark biochar, TiO_2_, and the combination of pine bark biochar with TiO_2_ (initial CEF concentration: 15 mg/L; adsorbent dosage: 150 mg/L; TiO_2_ conc. = 100 mg/L).

The kinetics of adsorption refers to the rate at which the sorption process takes place and is influenced by the interactions between the adsorbent and the adsorbate, as well as various system factors such as temperature, pH, initial concentration, and contact time. In the case of solid adsorbents, the mechanism can be broken down into three stages: mass transfer, which is the movement of the solute from the solution to the adsorbent’s surface at the solid–liquid interface; intraparticle diffusion, which describes the movement of the solute within the pores of the adsorbent; and adsorption, where the solute binds to the active sites on the adsorbent’s surface [54,55]. Adsorption kinetics refer to the rate at which the solute is adsorbed onto the adsorbent, which helps predict the rate of pollutant removal from the solution. Comprehending this rate is crucial for effectively designing and optimizing adsorption-based treatment processes. This experiment was conducted using pre-optimized pH values to achieve the most effective adsorption and to obtain more satisfactory results. The study of adsorption kinetics enables the evaluation of the rate at which CEF is removed from solution and allows the identification of potential limiting factors in the adsorption process. Kinetic analysis was performed by plotting adsorption efficiency versus time (Figure 5), providing essential insights into the intrinsic kinetics of the system, which depend on variables such as the type of adsorbent, temperature, and pH [2].

In all three experiments, a higher retention rate was observed during the first 15 min of the process, followed by a slower increase over time until reaching equilibrium or saturation of the biosorbent. The results demonstrate clear differences in the efficiency of CEF removal depending on the treatment applied. Pine bark biochar alone exhibited rapid and highly effective adsorption, achieving over 90% removal within the first 30–40 min and maintaining this performance throughout the experiment. In contrast, photoactivation with titanium dioxide under UV-Vis irradiation resulted in a slower but progressive removal of the antibiotic, reaching approximately 75% after 120 min. The combined system of pine bark biochar with photoactivated titanium dioxide showed the highest efficiency, with more than 90% removal occurring within 20 min and nearly complete degradation (~95%) by 60 min. This synergistic effect can be attributed to the rapid adsorption capacity of biochar, which reduces CEF concentration in solution, while titanium dioxide simultaneously promotes photocatalytic degradation, thereby preventing biochar saturation and enhancing overall removal. The UV-Vis control without either biochar or titanium dioxide exhibited negligible CEF removal (around 12–14% after 120 min), confirming that light irradiation alone is insufficient to degrade the antibiotic. Together, these results highlight that while pine bark biochar is a highly efficient adsorbent and titanium dioxide offers moderate photocatalytic activity, their combination under UV-Vis irradiation provides the most effective strategy for CEF removal.

Titanium dioxide (TiO_2_) is widely recognized for its dual adsorptive and photocatalytic properties in antibiotic removal from water. Adsorption concentrates antibiotic molecules at the TiO_2_ surface, enhancing subsequent photocatalytic degradation. Upon irradiation with photons of energy equal to or greater than its band gap, TiO_2_ generates electron–hole pairs that produce reactive oxygen species (ROS), mainly hydroxyl (•OH) and superoxide (O_2_^−^) radicals, which oxidize antibiotic functional groups such as β-lactam rings and aromatic moieties, leading to progressive degradation [33]. However, TiO_2_ activity is limited to the UV region, and its performance depends on factors such as pH, pollutant concentration, and the presence of inorganic ions or organic matter. Modifications like doping, sensitization, or coupling with other semiconductors have been explored to extend its photoresponse into the visible spectrum and reduce charge recombination [33,34]

In this study, CO_2_-activated pine bark biochar demonstrated strong potential as a sustainable and efficient adsorbent for CEF removal. When combined with TiO_2_ under UV–Vis irradiation, a synergistic effect was observed, improving both adsorption kinetics and overall removal efficiency (>95%). This synergy arises from biochar’s ability to enhance pollutant concentration near active sites and facilitate electron transfer during photocatalysis. Comparable studies, such as Nezhad et al. [35] and Usman et al. [36], reported high CEF removal using TiO_2_-based composites, particularly 93.7% removal at 400 mg/L using TiO_2_/MgO nanoparticles, and 96.52% degradation at 500 mg/L under optimal UV conditions (100 mg TiO_2_, pH 8, 9 h irradiation) but at unrealistically high pollutant concentrations. The present results demonstrate similar efficiencies under more representative conditions, confirming that coupling TiO_2_ with CO_2_-activated pine bark biochar offers an environmentally friendly and cost-effective approach for antibiotic-contaminated water treatment

In this study, the adsorption kinetics were interpreted using the pseudo-first-order and pseudo-second-order models and complemented by the application of the Elovich and intraparticle diffusion models to achieve a comprehensive understanding of the adsorption dynamics.

Table 3 summarizes the kinetic parameters obtained from fitting the experimental data to the pseudo-first-order and pseudo-second-order models.

The adsorption kinetics of CEF onto the tested materials are best described by the pseudo-second-order model, suggesting that the process is mainly controlled by chemisorption mechanisms. The combination of pine bark biochar with TiO_2_ not only enhanced the maximum adsorption capacity but also significantly accelerated the adsorption rate. The adsorption kinetics observed in the studies were best described by the pseudo-second-order model, with R^2^ values ranging from 0.999 to 1. This high degree of correlation indicates that the pseudo-second-order model accurately reflects the adsorption behavior of the CEF antibiotic onto pine bark-based biochar. The same was reported for the adsorption of CEF using mesoporous copper oxide nanosphere where the pseudo-second-order model presented the best correlation [49]. Also a combined electrocoagulation and absorption process used by Noudeh et al. [51] reached similar conclusions. Overall PSO is the most used kinetic model to describe antibiotics adsorption onto biochar [42].

Table 4 presents the parameters obtained from the Elovich and intraparticle diffusion models. The results from the Elovich model suggest that chemisorption contributes significantly to the adsorption process, particularly for TiO_2_. The intraparticle diffusion analysis indicates that while diffusion within the pores occurs, it is not the sole mechanism controlling the adsorption kinetics. Surface adsorption remains the dominant process, especially for the combined biochar and TiO_2_ system.

The kinetic modeling results provide insight into the adsorption mechanisms of CEF on pine bark, TiO_2_, and the pine bark–TiO_2_ mixture. According to the Elovich model, pine bark exhibited a very high initial adsorption rate (a = 3.6 × 10^5^) with a relatively high desorption constant (b = 1.57), and a good correlation coefficient (R^2^ = 0.94), suggesting that chemisorption processes may play an important role in CEF uptake. TiO_2_ showed a lower a value (4.21) and b = 0.43, but the highest R^2^ (0.98), indicating that the Elovich model describes its adsorption behavior more accurately, although the overall adsorption intensity was weaker compared to pine bark. The pine bark–TiO_2_ composite showed an even larger a value (5.58 × 10^8^) and higher b = 1.78, and a the relatively high R^2^ (0.97) suggests that the Elovich model does not fit this system as well, likely due to the more complex surface interactions in the composite material.

The intraparticle diffusion model provides additional insight into the adsorption mechanism. Pine bark displayed the intercept (C = 9.60), suggesting significant boundary layer effects and surface adsorption, while its diffusion rate constant (kdiff = 0.180) was relatively low. TiO_2_, in contrast, exhibited a higher diffusion constant (kdiff = 0.658) and a lower intercept (C = 5.60), indicating that intraparticle diffusion contributed more significantly to the adsorption process. The mixture showed the highest intercept (C = 12.40) and the similar kdiff = 0.197 to biochar alone, pointing to dominant surface adsorption with very limited intraparticle diffusion.

Overall, these results suggest that pine bark relies mainly on surface adsorption with strong chemisorption tendencies, TiO_2_ exhibits more diffusion-controlled adsorption with a better Elovich model fit, and the composite, while showing high adsorption potential, presents a more complex mechanism that neither model describes perfectly.

A deeper understanding of the various mechanisms involved in antibiotic adsorption is of great importance for improving the efficiency of water treatment systems. Such knowledge is essential for optimizing the remediation of these pollutants and ensuring the preservation of the environment for future generations.

## 3. Materials and Methods

### 3.1. Adsorbent and Catalyst

The adsorbent used in this study was a biochar derived from pine bark. The biomass underwent pyrolysis at 450 °C, followed by thermal activation at 800 °C with a controlled heating rate of 10 °C per minute. The sample was maintained at the final temperature for 60 min to ensure complete activation. The activation atmosphere consisted of a continuous flow of carbon dioxide. All gas flow rates refer to standard temperature and pressure (STP) and were supplied as dry, high-purity gases. No steam or additional oxidants were introduced during the treatments. The estimated linear gas velocities under these conditions, calculated according to pressure, tube cross-section, and temperature, are consistent with typical values for a 1″ inner-diameter quartz tube. The furnace was pre-purged for approximately 30 min with nitrogen (200 mL min^−1^) to remove residual air, after which the flow was switched to CO_2_ and the temperature increased at 10 °C min^−1^ up to 800 °C, followed by a 120 min dwell at this temperature. During both the heating and holding stages, the CO_2_ flow was maintained at ~150 mL min^−1^ (STP), corresponding to approximately 100–200 mL min^−1^ g^−1^ of biochar. Cooling was performed under a continuous nitrogen atmosphere to prevent oxidation.

TiO_2_ (TiO_2_, Aeroxide^®^ P25, Evonik Industries AG, Essen, Germany) and pine bark biochar were dosed at their individually optimized loadings because they operate under different rate-limiting mechanisms (photocatalysis vs. adsorption). For P25, excessive solids promote light scattering/self-shielding and agglomeration, leading to a non-monotonic activity–loading relationship; for biochar, performance is governed by available adsorption sites and mass transfer. Accordingly, all comparative analyses are reported with normalized metrics—for photocatalysis, removal expressed per gram of catalyst (mg g^−1^) (and where applicable, kinetic parameters normalized by catalyst mass); for adsorption, removal per gram of adsorbent (mg g^−1^) under the stated conditions. For adsorption and photocatalytic assays, a physical co-suspension (slurry) was prepared directly in the reaction flask by adding pine bark biochar and TiO_2_ (P25, Evonik) at their individually optimized loadings. In the combined system, concentrations of 150 mg L^−1^ biochar and 100 mg L^−1^ TiO_2_ (P25) were employed, while single-material control tests until homogeneous prior to whole simultaneous process (TiO_2_ + Biochar) testing. Adsorption experiments were conducted under dark conditions to ensure equilibrium before illumination, whereas photocatalytic runs were carried out using the same co-suspension under UV irradiation. No impregnation, deposition, or chemical anchoring procedures were applied at any stage.

### 3.2. Antibiotic

**Figure 6:**
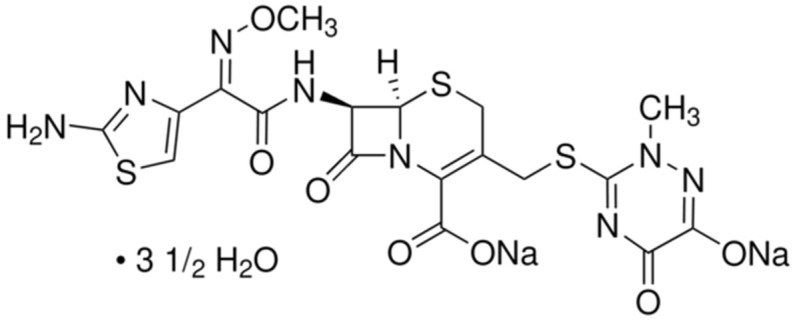
Chemical structure of CEF.

The antibiotic selected for this study was ceftriaxone sodium (CEF), a third-generation semi-synthetic cephalosporin provided by a pharmaceutical company. The antibiotic was used in the form of disodium hemieptahydrate, illustrated in Figure 6.

CEF appears as an almost white to pale yellow, slightly hygroscopic crystalline powder, being easily soluble in water, moderately soluble in methanol, and poorly soluble in anhydrous ethanol. A stock solution of CEF was prepared by dissolving the compound in distilled water to achieve a concentration of 15 mg/L.

### 3.3. Preparation of Adsorbent Systems for Isotherm and Kinetic Studies

For adsorption and kinetics experiments, the following conditions were applied:

– Pine bark biochar: 150 mg/L;
– Titanium dioxide (TiO_2_): 100 mg/L.

Photocatalysis was performed by exposure to UV-Vis in solar chamber (500 W/m^2^). Photocatalytic experiments were carried out in a bench-top xenon solar chamber (SUNTEST^®^ CPS+, Atlas Material Testing Technology LLC, Mount Prospect, IL, USA) equipped with an air-cooled 1500 W xenon lamp. The chamber is fitted with Daylight/Window Glass filters and an additional UVC cutoff filter, in compliance with ISO 4892-2 standards [56], providing a controllable irradiance within the 300–800 nm spectral range. The irradiance at the reactor plane typically ranged from ≈250 to 765 W m^−2^, with high spatial uniformity across the illuminated area (≈560 cm^2^). The output intensity was regulated using the instrument’s internal controller and verified externally with a calibrated radiometer (ILT1400A, International Light Technologies Inc., Peabody, MA, USA) at five measurement points (center and quadrants) to confirm uniformity and temporal stability (<5% drift). Prior to each experiment, the lamp was stabilized for at least 15 min to ensure consistent light output. A schematic representation of the optical layout and the reactor position within the chamber is provided in Figure S3.

The pH optimization was performed exclusively for the pine bark biochar system, as its surface functional groups are strongly affected by protonation–deprotonation equilibria, significantly altering adsorption efficiency. In contrast, TiO_2_ (TiO_2_, Aeroxide^®^ P25, Evonik Industries AG, Essen, Germany), an inorganic oxide with stable surface hydroxyls, shows minimal adsorption across the studied pH range [57].

The influence of pH on CEF adsorption was evaluated within the range pH 2–8, adjusted with dilute HCl or NaOH. Adsorption data at these discrete pH values are presented in Figure 1. Based on these results, the pH values 3.0 ± 0.1, 4.0 ± 0.1, and 6.0 ± 0.1 were selected for detailed kinetic and isotherm experiments. Experiments were conducted in triplicate using 200 mL of antibiotic solution at 15 mg/L and 150 mg/L of biochar. The samples were stirred continuously in a shaker at room temperature for 60 h. After agitation, the solutions were filtered by gravity, and the residual antibiotic concentration was measured by UV-visible spectrophotometry.

An additional kinetic study was performed to assess adsorption rates at pH 3, 4, and 6, with a contact time of 120 min.

### 3.4. Study of Equilibrium Isotherms

The Langmuir isotherm model assumes that adsorption occurs as a monolayer onto a homogeneous adsorbent surface, where all adsorption sites are identical and can be occupied by only one adsorbate molecule. It further presumes the absence of interactions between adsorbed species [42]. The linearized form of the Langmuir adsorption isotherm equation is expressed by Equation (1).

(1) $$\frac{C_{e}}{q_{e}}=\frac{1}{K_{L}q_{m}}+\frac{C_{e}}{q_{m}}$$

where $C_{e}$ (mg/L) is the equilibrium concentration, $q_{e}$ (mg/g) is the amount adsorbed at equilibrium, $K_{L}$ (L/mg) is the Langmuir constant related to the affinity between the sorbent and the sorbate, $q_{m}$ (mg/g) represents the maximum monolayer adsorption capacity.

The Langmuir constant $K_{L}$ indirectly reflects the free energy of adsorption, while $q_{m}$ is a valuable parameter for comparing the adsorption capacities of different materials.

To assess the favorability of the adsorption process, the dimensionless equilibrium $R_{L}\;$was calculated according to the following Equation (2).

(2) $$R_{L}=\frac{1}{1+K_{L}C_{0}}$$

where $C_{0}$ (mg/L) is the initial concentration. The nature of adsorption can be classified based on the value of $R_{L}$ as irreversible ($R_{L}$ = 0), favorable (0 < $R_{L}$ < 1), linear ($R_{L}$ = 1), or unfavorable ($R_{L}$ > 1) [58].

In contrast, the Freundlich isotherm describes adsorption onto heterogeneous surfaces, allowing multilayer adsorption and considering interactions between adsorbed molecules [59]. The linearized form of the Freundlich isotherm is given by Equation (3).

(3) $$\ln(q_{e})=\ln(K_{f})+\frac{1}{n}\ln C_{e}$$

where $K_{f}$ is the Freundlich constant indicative of adsorption capacity, $\frac{1}{n}$ is a heterogeneity factor, with *n* related to the adsorption intensity.

The value of $\frac{1}{n}$ provides insights into the favourability of the process [57]:

– Irreversible adsorption ($\frac{1}{n}\;$= 0);
– Favorable adsorption (0 < $\frac{1}{n}<1)$;
– Unfavorable adsorption $(\frac{1}{n}>1$).

The parameters derived from these models allow for a deeper understanding of the adsorption mechanisms and surface properties of the sorbent materials studied.

### 3.5. Study on the Equilibrium Adsorption of Antibiotics

The batch method is commonly employed in equilibrium studies owing to its simplicity and operational efficiency. In this study, CEF solutions with a concentration of 15 mg/L were prepared in 200 mL volumetric flasks and maintained at room temperature under continuous stirring using a magnetic stirrer. Following the contact/agitation period, 30 mg of sorbent was added to each solution. The mixtures were then subjected to continous stirring for 15, 30, 45, 60, and 120 min, respectively. The remaining concentration of the antibiotic in the solutions was determined using UV-visible spectrophotometry.

The amount of antibiotic adsorbed at time *t* ($q_{t})\;$and at equilibrium ($q_{e})\;$was calculated by this Equations (4) and (5), respectively, and are expressed in mg/g.

(4) $$q_{t}=\frac{\left(C_{0}-C_{t}\right)\times V}{m}\;\;$$

(5) $$q_{e}=\frac{\left(C_{0}-C_{e}\right)\times V}{m}\;\;$$

where $C_{0}$, $C_{t}$ and $C_{e}$ are the initial, time-dependent, and equilibrium concentrations (mg /L); *V* is the volume (L); and *m* the mass of sorbent (g).

Equation (6) was used to calculate the degree of adsorption R (%).

(6) $$R\left(\%\right)=\frac{\left(C_{0}-C_{t}\right)}{C_{0}}\times 100\;$$

### 3.6. Study of Adsorption Kinetics

In the pseudo-first-order kinetic model, the rate at which active adsorption sites on the sorbent are occupied is directly proportional to the number of unoccupied sites, as shown in this Equation (7).

(7) $$\ln(q_{e}-q_{t})=\ln q_{e}-k_{1}t$$

where

– $q_{e}$ (mg/g) is the amount of solute adsorbed at equilibrium;
– $q_{t}$ (mg/g) is the amount adsorbed at time *t*;
– $k_{1}$ (min^−1^) is the pseudo-first-order rate constant, representing the rate at which adsorption sites are occupied.

Higher $k_{1}$ values indicate a faster adsorption process.

Adsorption is described as the interaction between the adsorbate and two independent vacant sites on the adsorbent, in accordance with the pseudo-second-order kinetic model. The linear form of this model is represented by Equation (8).

(8) $$\frac{t}{q_{t}}=\frac{1}{k_{2}q_{e}^{2}}+\frac{t}{q_{e}}$$

In this model, $k_{2}$ represents the pseudo-second-order adsorption rate constant (g/mmol·min). Using the rate constant $k_{2}$, the initial adsorption rate *h* (mg/g·min) is defined by Equation (9).

(9) $$h=k_{2}\times q_{e}^{2}$$

The Elovich equation was proposed by Roginsky and Zeldovich in 1934 [55], Equation (10).

(10) $$q_{t}=(\frac{1}{b})\;\ln(ab)+(\frac{1}{b})\;ln(t)$$

where

– *a* (mg/g·min) represents the initial adsorption rate;
– *b* (g/mg) is related to the extent of surface coverage and activation energy for chemisorption.

Lower *b* values suggest higher activation energy and slower adsorption.

The intraparticle diffusion kinetic model equation (Equation (11)) was introduced by Weber and Morris in 1963 to describe the diffusion of the adsorbate within the pores of the adsorbent.

(11) $$q_{t}=k_{t}t^{\frac{1}{2}}+C$$

where

– $k_{t}$ (mg/g·min^1/2^) is the intraparticle diffusion rate constant, indicating the rate at which the solute diffuses inside the pores;
– *C* (mg/g) is the intercept, which gives an idea about the thickness of the boundary layer; a larger *C* suggests a greater boundary layer effect.

If a plot of *q_t_* versus $t^{\frac{1}{2}}$ is linear and passes through the origin (C ≈ 0), intraparticle diffusion is the sole rate-limiting step.

### 3.7. Biochar Characterization

A comprehensive characterization of the biochar samples was carried out through several complementary techniques, including X-ray diffraction (XRD), scanning electron microscopy (SEM), nitrogen physisorption (BET analysis), and Fourier-transform infrared spectroscopy (FTIR). The textural properties were evaluated from N_2_ adsorption–desorption isotherms obtained at −196 °C and from CO_2_ adsorption measurements at 0 °C. The use of CO_2_ as carrier gas did not lead to noticeable variations in the results. The TiO_2_ employed in this work corresponds to the commercial P25 material (Evonik–Degussa), a benchmark photocatalyst widely used in environmental applications. Since this material has been thoroughly characterized in numerous studies, its detailed physicochemical properties are not repeated here to avoid redundancy. Previous works have comprehensively described its crystal structure, morphology, electronic properties, and photocatalytic performance [33,60,61]

XRD patterns were collected using a Philips X’Pert Pro diffractometer (PANalytical B.V., Almelo, The Netherlands) equipped with a θ–2θ goniometer, Cu Kα radiation (λ = 1.54053 Å), and an X’Celerator detector. FTIR analyses were performed in diffuse reflectance mode (DRIFT) on a JASCO FT/IR–6200 IRT-5000 (JASCO Corporation, Tokyo, Japan) spectrometer. The specific surface area and pore characteristics were derived from adsorption data using a Micromeritics ASAP 2010 analyzer (Micromeritics Instrument Corp., Norcross, GA, USA). The surface morphology and microstructural features were examined with a Hitachi S-4800 field-emission SEM coupled with a Bruker X-Flash 4010 EDX detector for elemental analysis (Hitachi High-Technologies Corp., Tokyo, Japan; Bruker Corporation, Billerica, MA, USA).

In addition to the structural and chemical characterization, the point of zero charge (pHₚzc) was also determined to better understand how the surface charge of the biochars varies with pH.

## 4. Conclusions

This study demonstrated the potential of CO_2_-activated pine bark biochar as an effective and sustainable adsorbent for the removal of CEF from aqueous solutions. The biochar exhibited favorable physicochemical properties, including a high specific surface area (583 m^2^/g) and a well-developed microporous–mesoporous structure, which enhanced its adsorption capacity and facilitated multiple sorption mechanisms. These properties, combined with its environmentally friendly origin and low cost, make pine bark biochar a promising material for environmental remediation.

The adsorption efficiency was shown to be strongly pH-dependent, with maximum CEF removal (>95%) occurring at acidic conditions around pH 3, due to the electrostatic attraction between the anionic antibiotic species and the positively charged biochar surface below its point of zero charge (pHpzc = 6.7). Kinetic studies revealed that adsorption was rapid within the first minutes of contact and best described by the pseudo-second-order model, confirming that chemisorption plays a dominant role. The Elovich and intraparticle diffusion models further indicated that adsorption proceeds through surface-controlled mechanisms with contributions from diffusion processes, particularly for TiO_2_.

Isotherm modeling showed that CEF adsorption onto pine bark biochar, TiO_2_, and their combination followed predominantly Langmuir-type behavior, consistent with monolayer adsorption on homogeneous surfaces. Among the tested systems, TiO_2_ achieved the highest maximum adsorption capacity (280 mg/g), while pine bark biochar reached 154 mg/g, a value higher than that of many biochars reported in the literature. Importantly, the synergistic combination of biochar with TiO_2_ under UV–Vis irradiation significantly improved both adsorption kinetics and overall removal efficiency (>95%), highlighting the complementarity between rapid adsorption and photocatalytic degradation.

Overall, the findings confirm that pine bark biochar is a sustainable and efficient adsorbent for pharmaceutical contaminants, and its performance can be further enhanced through integration with photocatalytic materials such as TiO_2_. This combined adsorption–photocatalysis approach offers a promising pathway for the treatment of antibiotic-polluted waters, balancing cost-effectiveness, environmental safety, and high efficiency. Future work should focus on optimizing operational parameters, testing in real wastewater matrices, and evaluating the long-term stability and regeneration potential of the biochar–TiO_2_ system.

## Figures and Tables

**Table 1:** Physicochemical Properties of CO_2_-Activated Biochar.

Parameter		CO_2_-Activated Biochar
XRD	Crystalline Phases	Presence of CaCO_3_
SEM	Surface Morphology	Highly porous
BET	Specific Surface Area (m^2^/g)	583
	Pore Structure	Micropores and mesopores
FTIR	Oxygenated Groups	Significant loss
Point of Zero Charge	pHpzc	6.7

**Table 2:** Isotherm parameters of CEF adsorption onto the different methods.

		Langmuir			Freundlich		
	R^2^	q_max_ (mg/g)	K_L_ (L/mg)	R_L_ (L/mg)	R^2^	1/n	K_F_ (mg/g)
Pine Bark	0.999	153.504	1.434	1.38 × 10^−2^	0.935	0.20	83.9
TiO_2_	0.988	279.619	0.427	4.47 × 10^−2^	0.984	0.36	96.8
Pine Bark + TiO_2_	0.994	216.982	0.904	2.16 × 10^−2^	0.961	0.26	91.2

**Table 3:** Parameters for the kinetic sorption data using pseudo-first-order and pseudo-second-order kinetic models.

Biosorbent	Pseudo-First-Order Model				Pseudo-Second-Order Model				
	k_1_ (min^−1^)	q_e_ calc (mg/g)	q_e_ exp (mg/g)	R^2^	k_2_ (g/(mg·min))	h (mg/(g·min))	q_e_ calc (mg/g)	q_e_ exp (mg/g)	R^2^
Pine Bark	−0.048	2.773	11.315	0.987	0.036	4.824	11.574	11.315	1.000
TiO_2_	0.052	13.673	12.150	0.951	0.006	1.074	13.532	12.150	0.999
Pine Bark + TiO_2_	0.073	2.295	13.855	0.957	0.093	18.215	13.966	13.855	1.000

**Table 4:** Parameters for the kinetic data using Elovich and intraparticle diffusion models.

Biosorbent	Elovich Model			Intraparticle Diffusion Model		
	a	b	R^2^	C	k_diff_	R^2^
Pine Bark	3.6 × $10^{5}$	1.57	0.940	9.60	0.180	0.854
TiO_2_	4.21	0.43	0.977	5.60	0.658	0.911
Pine Bark + TiO_2_	5.58 × 10^8^	1.78	0.967	12.40	0.197	0.923

## Data Availability

The original contributions presented in this study are included in the article/Supplementary Material. Further inquiries can be directed to the corresponding author.

## Supplementary Materials

The following supporting information can be downloaded at: https://www.mdpi.com/article/10.3390/molecules30214291/s1, Figure S1: Biochar FTIR; Figure S2: Biochar SEM; Figure S3: Solar chamber Suntest Atlas CPS+; Figure S4: XRD patterns of biochar.
