# Supplementary material for: Kinetics and Isotherm Study of Ceftriaxone Removal Using Functionalized Biochar Combined with Photocatalysis
Source: Molecules. 2025 Nov 5;30(21):4291. doi: 10.3390/molecules30214291 (PMC12608820; doi:10.3390/molecules30214291)
Supplement: Supplementary file 1 [file molecules-30-04291-s001.zip › molecules-3935597-supplementary.pdf]

Supplementary Material

Kinetics and Isotherm Study of Ceftriaxone Removal Using Functionalized Biochar Combined with Photocatalysis

Luísa Cruz-Lopes [1], Rodrigo Araújo [2], Ana Rita Lopes [3], Samuel Moles [4,5], Francisca Romero-Sarria [6] and Bruno Esteves [1,*]

1 CERNAS (Centre for Natural Resources, Environment and Society)-IPV Research Centre, Polytechnic University of Viseu, Av. Cor. José Maria Vale de Andrade, 3504-510 Viseu, Portugal; lvalente@estgv.ipv.pt
2 Department of Environmental Engineering, Polytechnic University of Viseu, Av. Cor. José Maria Vale de Andrade, 3504-510 Viseu, Portugal; rodrigokenzo2202@gmail.com
3 Faculty of Dental Medicine, Universidade Católica Portuguesa, 3504-505 Viseu, Portugal; s-arvlopes@ucp.pt
4 Instituto de Investigación en Ciencias Ambientales de Aragón (IUCA), Universidad de Zaragoza, c. de Pedro Cerbuna, 12, 50009 Zaragoza, Spain; sma@unizar.es
5 Department of General Chemistry, Escuela Universitaria Politécnica La Almunia Universidad de Zaragoza, C. Mayor, 5, 50100 La Almunia de Doña Godina, Zaragoza, Spain
6 Department of Inorganic Chemistry and Institute of Materials Science, Joint Center University of Seville-CSIC, Av. Américo Vespucio, 41092 Seville, Spain; francisca@us.es
* Correspondence: bruno@estgv.ipv.pt

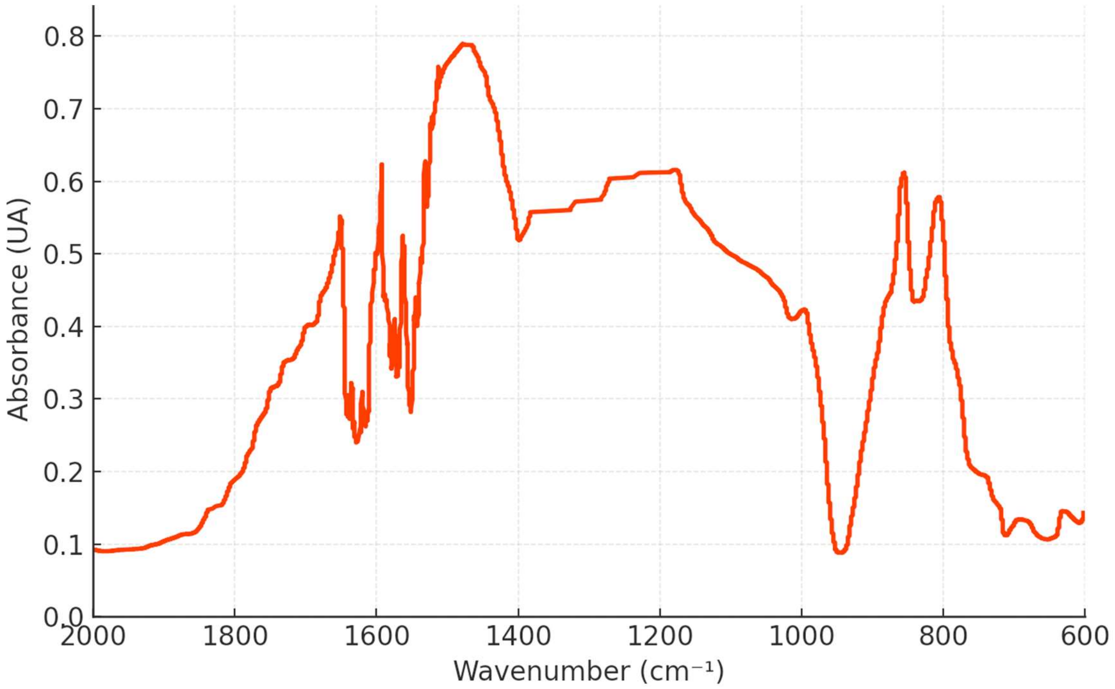

Figure S1. Biochar FTIR

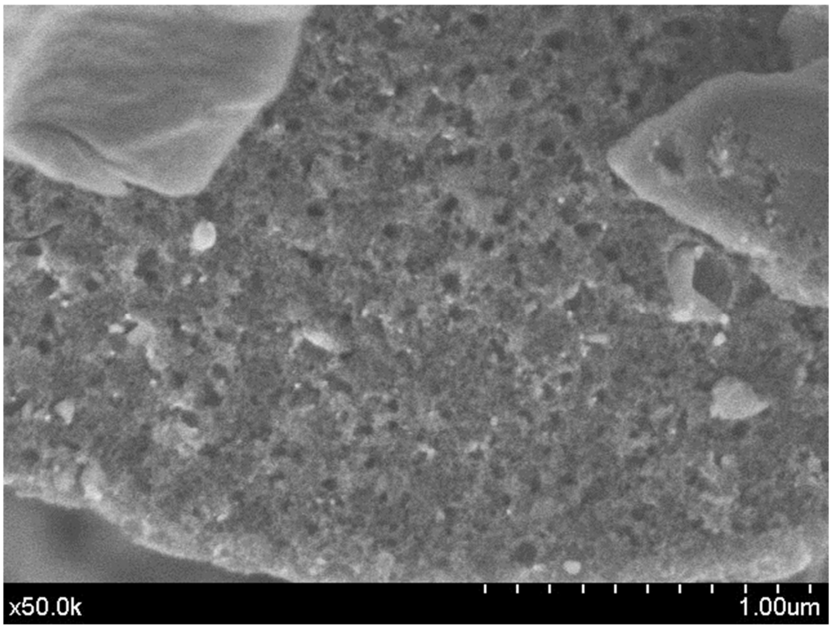

Figure S2. Biochar SEM

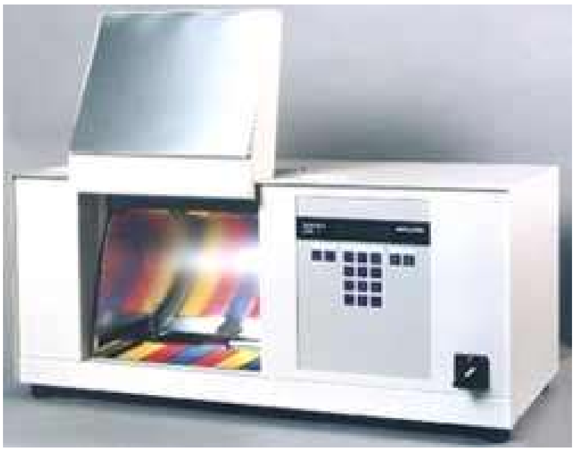

Figure S3. Solar chamber Suntest Atlas CPS+

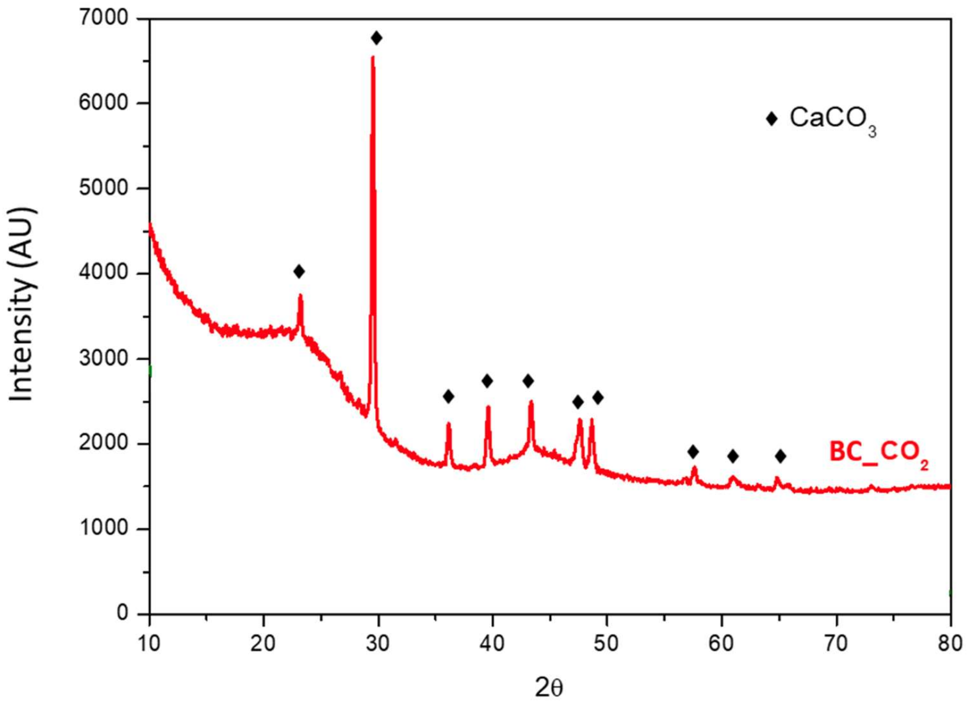

Figure S4. XRD patterns of biochar
